# Perceptual Matching of Room Acoustics for Auditory Augmented Reality in Small Rooms - Literature Review and Theoretical Framework

**DOI:** 10.1177/23312165221092919

**Published:** 2022-05-03

**Authors:** Annika Neidhardt, Christian Schneiderwind, Florian Klein

**Affiliations:** 1 26559 Technische Universität Ilmenau, Germany

**Keywords:** Augmented Reality, perception, room Acoustics, binaural Synthesis, 6DOF, small Rooms

## Abstract

For the realization of auditory augmented reality (AAR), it is important that the room acoustical properties of the virtual elements are perceived in agreement with the acoustics of the actual environment. This perceptual matching of room acoustics is the subject reviewed in this paper. Realizations of AAR that fulfill the listeners’ expectations were achieved based on pre-characterization of the room acoustics, for example, by measuring acoustic impulse responses or creating detailed room models for acoustic simulations. For future applications, the goal is to realize an online adaptation in (close to) real-time. Perfect physical matching is hard to achieve with these practical constraints. For this reason, an understanding of the essential psychoacoustic cues is of interest and will help to explore options for simplifications. This paper reviews a broad selection of previous studies and derives a theoretical framework to examine possibilities for psychoacoustical optimization of room acoustical matching.

## Introduction

Binaural technology attempts to mimic acoustical cues which are relevant for spatial hearing (Blauert,^1997^). It aims at creating spatial auditory illusions that are in agreement with the listener’s expectations. Auditory illusions are interesting for the realization of Virtual Reality (VR), Augmented Reality (AR) and Mixed Reality (MR). Definitions of these terms vary among the literature (Wu et al.,^2013^). VR describes fully virtual environments, which do not correspond to the user’s real environment (Novo,^2005^). According to the Reality-Virtuality continuum described by Milgram et al. (1995), AR is defined as a subset of MR where virtual content is added to the real environment. This is contrary to Augmented Virtuality, also a subset of mixed reality, where real world objects are integrated with the virtual environment. This review deals with the perceptual interaction between added virtual acoustic objects and the real environment, which we refer to as AR.

For the realization of augmented auditory reality (AAR), the virtual acoustic objects have to be seamlessly integrated into the real environment. This requires to match the acoustical properties of the virtual elements with the real environment. The occurrence of room reflections influences the auditory appearance of a sound source, for example, its apparent source position, sound level or source width. The minimum target of AAR is to induce a *plausible* auditory illusion. Plausible illusions are perceived in agreement with an internal reference that people develop based on their listening experience from everyday life (Kuhn-Rahloff,^2012^). For convincing virtual versions of real acoustic objects it is desired that they cannot be identified as virtual. Revealing perceptual cues have to be minimized. *Authentic* auditory illusions are perceived in agreement with the external reference (Brinkmann et al.,^2017^). This means, they are perceptually identical to their real counterparts.

For the development of suitable room matching approaches, the following questions arise:

What are the requirements to achieve plausibility?
How do listeners notice that a sound source is not real?
What are revealing factors?
How does a mismatch of room acoustics contribute?
What is the perceptually required physical accuracy?
Is it possible to achieve authenticity with the given practical constraints?

A high-quality realization of an AAR scenario with minimum computational effort relies on a detailed understanding of the factors contributing to the perception of room acoustics. This paper examines the perceptual requirements for the realization of AAR by a literature review and derives a theoretical framework. The discussion focuses on *small* rooms like classrooms, living rooms, or offices due to their high practical relevance as common environments for augmented reality applications. According to Kleiner & Tichy (2014), small rooms have a volume of up to a few hundred $m^{3}$ . At this size, room modes are perceptually more relevant, early reflections arrive with shorter delays, the echo density rises more quickly than in concert halls, and smaller distances to the sound source as well as reflecting objects are common. Furthermore, AAR often addresses scenarios wherein the listener walks around instead of being seated in an audience in a stable distance to the sound source. The listener may get close to the sound source, close to walls or other reflectors, and walk around sound sources and behind. Spatial auditory illusions have to endure a listener’s motion in six degrees of freedom (6DOF) in order to be convincing.

AAR is still an emerging interdisciplinary and complex field, although numerous studies have been conducted already. Combining their results and analyzing their interrelations will provide new insights and allow for drawing new conclusions. This mapping review (Grant & Booth, 2009) is an attempt to structure, categorize and summarize, what is known so far with the goal to create map of current status of knowledge in this field. In addition, new research questions will be derived.

**Figure 1.**
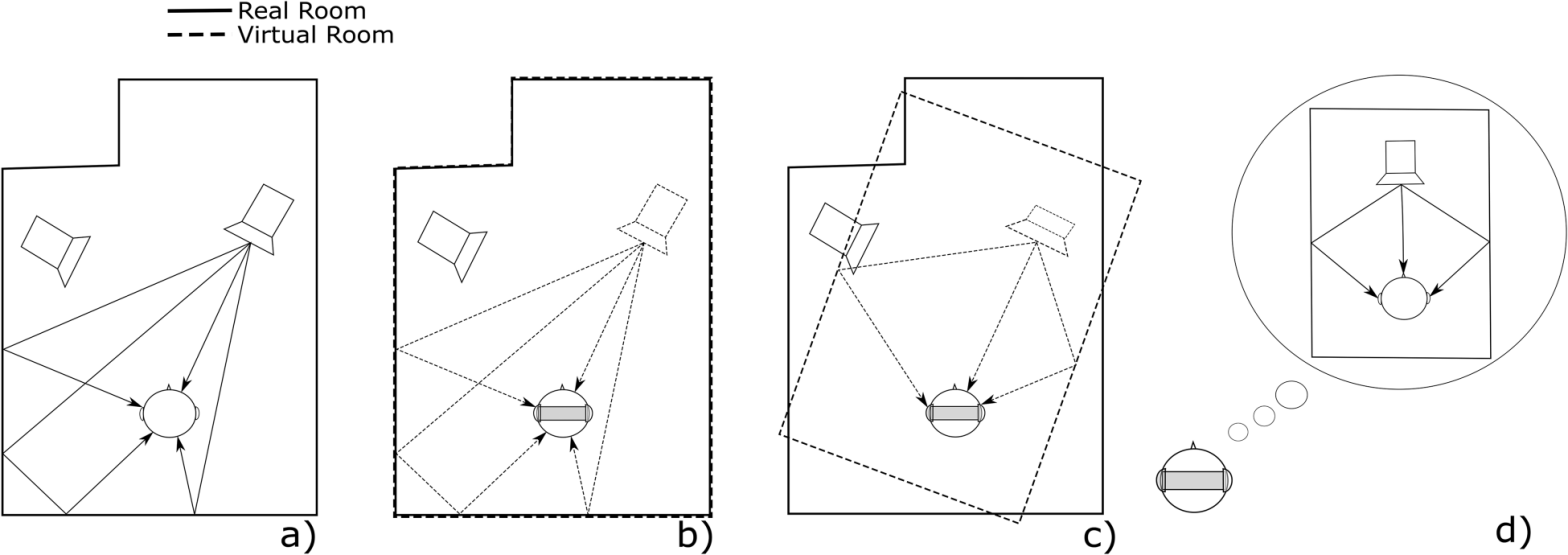
This article presents the concept of perceptual matching of room acoustics for AAR with - a) the original real version of a sound source in a room - b) a physically perfectly matched virtual room, which is hard to achieve in real applications - c) a physically slightly different, but perceptually matched room - d) the listener’s expectation which may be different from the original sound field.

Figure 1 illustrates our basic idea of perceptual room matching. A physically perfect imitation (Figure 1b) of a real sound object in a given environment (Figure 1a) would lead to exactly the sound pressure at the listener’s ear drums. In practice, this reconstruction of the sound pressure is subject to technical limitations, such as real-time constraints, limited processing power and incomplete information about the given environment. Therefore, a simplified approximation of a room’s acoustic properties (Figure 1c) that still satisfy the listener’s perceptual demands (Figure 1d) are of interest. The allowed physical deviation of c) from b) is determined by the accuracy and tolerances of the listener’s expectations illustrated in d).

Currently, there is no consensus in the research community on the best strategy to mimic a correct room acoustic perception with a minimal set of determined cues. This means that the most suitable combination of a reproduction method and the estimation of the most essential parameters of the surrounding sound field has not yet been identified. At present, there are a large variety of approaches, study designs, and research questions. This lack of common procedures and evaluation methods provides only a few options for comparing between different studies so far. To give a comprehensive overview, we structured this article as follows. As a starting point, an overview of a basic technical system for AAR is provided, followed by a general summary on perceptual similarity of different rooms. The first main section discusses the formation of the listener’s expectations as a foundation for the perceptual requirements. The second main section reviews studies that contribute to determining the corresponding physical requirements for the technical realization. This second part is structured according to features of a (binaural) room impulse response. These two main parts are followed by an analysis of how to measure the success of perceptual room matching and finally, the conclusions.

## Basic Technical System for Auditory Augmented Reality

One straightforward approach to realize an AAR scenario is to use a headphone-based binaural reproduction system. Dry mono signals are convolved with binaural room impulse responses (BRIRs), which contain the room acoustic and head-related cues to create an externalized spatial auditory impression (Blauert, 1997). For AAR, the auditory reproduction is synthesized considering information about acoustical properties acquired from the environment. This can be done either a priori, for example, by pre-measurements and offline pre-processing, or in real-time “on-the-fly”. Over the years, a variety of approaches to synthesize binaural room impulse responses for a 6DOF listening area have been presented. These are all built on (a small amount of) a priori information about the room and the sound sources within. The available information can be acoustic impulse responses, measured with a single (omni-directional) microphone (Pörschmann et al., 2017), a head-and-torso-simulator (Sloma et al., 2019; Garcia-Gomez & Lopez, 2018) or microphone array solutions (Garí et al., 2019; Stade, 2018; Zaunschirm et al., 2020; Müller & Zotter, 2020; McCormack et al., 2020; Engel & Picinali, 2022). Besides, for example, semantic and visual information can be used to estimate acoustic properties (Kim et al., 2017, 2020). BRIR synthesis can be realized either by pure simulation, for example, based on ray-tracing (Savioja & Svensson, 2015; Brinkmann et al., 2019), wave-based simulation approaches or delay networks (Alary et al., 2019; Välimäki et al., 2012) or by manipulation of measured impulse responses, like interpolation (Bruschi et al., 2020; Brinkmann et al., 2020), extrapolation (Neidhardt et al., 2018; Sloma et al., 2019; Coleman et al., 2017; Pörschmann et al., 2017) or shaping of the late reverberation tail (Jot & Lee, 2016; Pörschmann & Zebisch, 2012; Arend et al., 2021). Systems that do not rely on a priori knowledge are desired because their use is not limited to rooms or environments for which the predetermined information is available. Such systems attempt to analyze the listener’s current environment in (close to) real-time, based on streamed microphone signals or/and signals from other types of sensors and adjust the reproduction accordingly. Depending on the complexity of the capturing system and the desired level of detail, the computational effort of the scene analysis can be quite high and may not meet (close to) real-time requirements. Sophisticated and quite robust approaches for the blind estimation of the room impulse response (Crocco & Del Bue, 2015), reverberation time (RT), or direct-to-reverberant-energy-ratio (DRR) have been proposed (Eaton et al., 2016). RT or early-to-late-energy-ratios (ELR) can be estimated for broad or selected frequency ranges (Xiong et al., 2018; Li et al., 2019). This is, for example, used for automatic speech recognition and the necessary dereverberation.

For AAR, a targeted combination of these approaches with established methods for binaural rendering is desired. Which acoustical parameters are relevant? In this contribution, we present a review of studies investigating possible key elements for a successful adaptation of the reproduced room acoustics to the real environment. Not all elements have been understood in detail yet. One of the challenges is the complex and multi-modal nature of room perception, which is also subject to cognitive effects. In order to create an efficient realization capable of adjusting the reproduction in (close to) real-time, a psychoacoustic optimization of both scene analysis and spatial audio rendering is inevitable. This demands understanding the contribution of the single physical parameters and the required accuracy, which still generates spatial auditory illusions of the desired quality.

## Perception of Room Acoustics and Perceived Acoustical Similarity

In comparison to free-field conditions, in which only the direct sound of a sound source arrives at the listener, the occurrence of additional reflections may increase the apparent sound level, affect the apparent source width, vary the apparent source position in direction and especially in distance, may cause deviations in the perceived timbre and increase reverberance (Kuttruff, 2017, p. 163). The perceptual effects of room acoustics have been studied in various areas of research, highlighting the multidisciplinarity (Zahorik, 2021). These areas include, for example, speech intelligibility, architectural acoustics, sound reproduction and echolocation. Room acoustics provide valuable information for spatial hearing, like distance perception and externalization as well as auditory scene analysis. In contrast, it can also impair source localization or speech understanding. In the context of studying the preferred acoustical properties of concert halls, Vorländer (2011) summarizes that “the three most important factors (loudness, reverberance and spatial impression) explain most of the statistical variance when comparing the acoustic conditions in auditoria.” He also points out that open questions remain, for example, regarding “the listener’s sensitivity to changes in a sound field regarding those subjective aspects.”

For quantifying and adjusting the perceived acoustical similarity of two rooms, the correlations between perceptual quality features and physical measures of room acoustics must be understood. These correlations have been subject to research for several decades, especially in the context of concert halls. As a result, various room acoustic parameters determined from the physical properties of the sound fields in such halls have been developed to describe and predict their perception. A selection of such parameters is summarized in standard ISO-3382-1 (2009). These include, for example, RT, early decay time (EDT), clarity indices (C80 and D50), sound strength (G) as well as the interaural cross-correlation coefficient (IACC). The just-noticeable difference (JND) is the minimum change of the parameter, which produces a noticeable variation in the sensory experience (Fechner, 1889). The standard provides concrete values for the JNDs of the listed room acoustic parameters. However, additional studies indicate that JNDs can vary considerably from the specified values and can depend on other conditions like the type of signal, the frequency content, or the absolute values of the parameters of interest (Klockgether & van de Par, 2016; Martellotta, 2010; Dorrego & Vigeant, 2018).

In their review on room acoustical parameters as predictors of room acoustic impression, Weinzierl & Vorländer (2015) conclude that after “more than 50 years of research on developing psychoacoustical measuring instruments for the concept of ’room acoustical impression”’ and “more than 100 years of research on the development of physical measures which could serve as technical predictors for these perceptual qualities”, “the state of the art is surprisingly unsatisfactory.” In another review, Bradley (2011) shows that it is still unclear how some of these parameters should be calculated best. For example, findings by Barron suggest averaging EDT values from 125 Hz to 2 kHz works best, whereas the ISO standard suggests a mid-frequency average. Bradley also points out that more research on JNDs and their complexity is needed as they are essential to understand the correlations of such measures to the perception.

In addition to the remaining open questions about objective measures for the perception of concert hall acoustics, it is also not sufficiently clear to which extent this knowledge is valid for the acoustics of small rooms. Standard ISO3382-2 (2008) describes a procedure to estimate the reverberation time in ordinary rooms. No other parameters are listed. Some aspects of the established parameters have been motivated by the properties of the human auditory sense, which is still the same in small rooms. However, physical conditions like typical listening positions concerning the sound source, the types of sound sources, the decay behavior, and the progress of echo density after excitation differ from performance rooms. Are the JNDs the same under these conditions?

Moreover, cognitive effects like becoming familiar with and adjusting to the room can play a role. The auditory room perception can vary, although the physical sound field remains the same (Brandenburg et al., 2020). Such effects might even dominate over the influence of physical details under certain conditions.

van Dorp Schuitman et al. (2013) point out that the auditory perception of room acoustics also depends on the type of the source signal. Hence, there is a general shortcoming in the idea of predicting the perception of room acoustics only from parameters estimated from room impulse responses since this approach does not take the type of signal into account. Instead, the authors propose a new concept of parameters determined from binaural recordings of the sound field in the room. The correlation with the perception of reverberance, clarity, apparent source width, and listener envelopment is better in most cases with this method.

In the context of loudspeaker reproduction in rooms, it is important to distinguish between the bass-frequency range in which room modes can cause standing wave behavior and the range of mid and high frequencies Toole (2017, p. 153–156). For the frequencies above the transition range, rooms cause changes in the timbral and spatial perception of a loudspeaker reproduction. Strong early reflections can cause audible comb filter effects, shift the perceived position and size of the image of the sound source. Room resonances can cause audible change in timbre as well. Later arriving reflections contribute to a sense of spaciousness or listener envelopment.

Studying how the acoustics of small rooms influence the perception of a multi-loudspeaker reproduction, Kaplanis et al. (2019) observed that the perceptual differences were based on two main dimensions. These can be characterized by the four perceptual constructs *reverberance, width & envelopment, proximity, and bass*.

Zahorik (2009) studied the perceptual similarity of rooms with 15 small-room auralizations based on measured and simulated BRIRs. He concluded that “when at-the-ear signal levels were held constant, the rooms differed along just two perceptual dimensions: one related to reverberation time ( $T_{60}$ ) and one related to interaural coherence ( IACC )” (Zahorik, 2009, p. 1). The study did not consider listener motion, and in each room, only one listening position approximately in the center of the room was taken into account.

So far, only a few studies address the challenge of perceptual room matching, the most critical room acoustic parameters, and their required accuracy. If the reproduced room does not match the actual room, this may impair the perceived externalization (Werner et al., 2017; Udesen et al., 2015), auditory distance perception (Gil-Carvajal et al., 2016) and plausibility (Neidhardt & Kamandi, 2022). While perfect physical matching of the room is technically hard to achieve, a sufficient perceptual similarity is of great importance for the creation of AAR. Thus, it is necessary to identify the relevant factors, understand their interrelations and determine the required accuracy of the contributing physical parameters. We summarize this under the umbrella term **Perceptual Room Matching**. In the following, selected aspects of perceived similarity and perceptual matching of room acoustics are discussed.

### Example: Auditory Perception of Room Size

When asking naive listeners about the auditory room perception, a standard answer is a description of the assumed room size. This indicates that even inexperienced listeners subconsciously assess the auditory room impression and abstract certain assumptions about the room’s size, geometry, and type. The impression of the room size is likely to play an essential role in the perceived agreement of virtual room acoustics with the real environment. There have been attempts to estimate the size, shape (Tukuljac et al., 2018; Kim et al., 2017) and volume (Shabtai et al., 2010; Genovese et al., 2019) of rooms blindly. However, the auditory perception of room size does not linearly depend on the room’s actual size. It is also influenced by the geometrical arrangement and the acoustic properties of the interior. The apparent size of a room is the result of a combination of several physical parameters. Which acoustic cues are used for auditory room size estimation and how they interact is still not fully understood.

Hameed et al. (2004) studied the relation between RT, DRR, and the perceived room size based on simulated room impulse responses with a 16-channel reproduction system placed in an anechoic room. RT values of 0.62 s, 0.73 s, and 0.83 s caused a subsequent increase of the perceived room size. Variation of the DRR between -23, -25, and -28 dB did not yield a change in the room size perception. The results of Larsen et al. (2008) suggest that these differences are below the JND for DRR.

Cabrera et al. conducted several experiments and concluded that the auditorily perceived room size relies more on room acoustic characteristics than the actual room size. The clarity index was shown to be a good predictor for the perceived room size (Cabrera et al., 2005; Cabrera, 2007a). “Judgments of room size appear to be mainly based on reverberation energy parameters, and the role of IACC remains unclear” (Cabrera, 2007b, p. 8). Increasing RT and decreasing C80 led to the impression of an increased room size (Cabrera, 2007a; Cabrera et al., 2005). In the particular case of judging the auditory room size via headphone-based binaural synthesis, the reverberance has a greater effect on the perceived room size than in the real sound field (Cabrera et al., 2006; Cabrera, 2007b).

Yadav et al. (2011) studied the perceived room size when exciting the room with self-created oral, so-called *autophonic* stimuli. Room size judgments correlated with parameters RT, G, which is in this context also called *room gain*, and clarity index C50.

Sadalla & Oxley (1984, p. 394) studied the “relationship between the shape and the perceived size of rectangular and square rooms.” “The results indicate a substantial illusion produced by rectangularity; more rectangular rooms consistently were estimated as larger than less rectangular rooms of equal size. This effect was independent of the viewing position of the observer”.

Kolarik et al. (2021) found an influence of the type of signal on the estimated room size. With speech, the room was perceived as significantly larger than with clicks or noise.

Larsson & Väljamäe (2007) investigated the perception of room size-based visual-only and audio-only representations as well as for an audio-visual impression. A virtualized visual impression of the room led to a smaller perceived room size, while in the audio-only presentation, listeners usually overestimated the size of the room. For the audio-visual combination, in medium and small rooms, the subjects achieved the most accurate estimation of room size. Regarding the relation to RT and C80, the observations in the audio-only condition were in agreement with Cabrera et al. (Cabrera, 2007a; Cabrera et al., 2005).

Does this mean that vice versa BRIRs with similar RT and C80 values lead to similar apparent room sizes? Further research is required. Moreover, rooms with similar apparent room sizes may not be perceived as matching, for example due to differences in timbre.

In common AR scenarios, the listener not only hears but also sees the actual environment, for example, the office or living room where the AR system is used. The user is confronted with an audio-visual impression of the actual room, which may induce a certain expectation towards the room acoustic properties of the virtual content. This raises the question of what contributes to the formation of this expectation.

## Formation of the Listener’s Expectation

A common scenario is that a user activates the AAR device after he already spent some time in that specific room. In this case, the user of an AAR system expects the virtual objects to blend in with the real environment seamlessly. This requires that the virtual acoustic objects have a similar late reverberation as the other sound sources in the room, have no conspicuous coloration, and appear at positions that are meaningful regarding their content. Furthermore, it is known that reverberation influences localization, especially for distance. Thus, an adequate matching of room acoustics is necessary to ensure correct localization. Moreover, when starting to move the head or change the position in the room, the user expects the virtual object to behave like a real one.

If the virtual elements imitate known real sound objects, a listener has a certain expectation regarding their acoustical properties from his everyday listening experience. This includes, for example, their size, shape, and directivity, as well as their behavior. Depending on the listening experience concerning the reproduced content, these expectations, also referred to as the *internal reference* (Kuhn-Rahloff, 2012), can be very accurate, but also quite vague or even wrong. Car experts, for example, may have more detailed expectations for certain types of cars. If the virtual car is created in front of the listener and can be explored in 6DOF, car mechanics are very likely to be more critical listeners since they walk around cars and pay attention to the different sounds a lot during their everyday activities. In contrast, for people who are not interested in the sound of cars, a quite rough approximation may perfectly fulfill their expectations and lead to an AAR experience they like. Similarly, room acousticians and sound engineers pay more attention to the details of room acoustics in their everyday lives and are more likely to notice inaccuracies in the reproduced room (von Berg et al., 2021). Another interesting group are blind people, who are often assumed to have better hearing capabilities than people with normal vision. More and more studies indicate that blind people’s improved accuracy in auditory estimation skills like the localization of reflectors or determination of wall material (Kolarik et al., 2014; Thaler & Goodale, 2016), are due to more training and experience and cannot be observed for all blind people. For selected tasks, it was shown that people with normal vision could be trained to achieve similar accuracy as blind people (Teng & Whitney, 2011) and, for example, localize a reflector with similar accuracy as sound sources (Wallmeier et al., 2013). According to Thaler (Thaler, 2013) less than 30% of the blind people use echolocation on a regular basis, still with varying degrees of experience. Trained echolocators can localize reflectors in distances of several meters (Kolarik et al., 2014) based on small direction- and position-dependent variations of level and timbre of the sound. During these tasks, their brains showed activity in areas that are usually used for vision (Thaler & Goodale, 2016).

Generally, for AR systems, this means that the listener’s expectations, and thus the perceptually required level of acoustic detail to fulfill these expectations, depends on the target user group, their level of training and experience, the content, and the use case. This also needs to be considered in perceptual evaluations of such systems. It is incorporated in the further discussion within this review.

**Figure 2.**
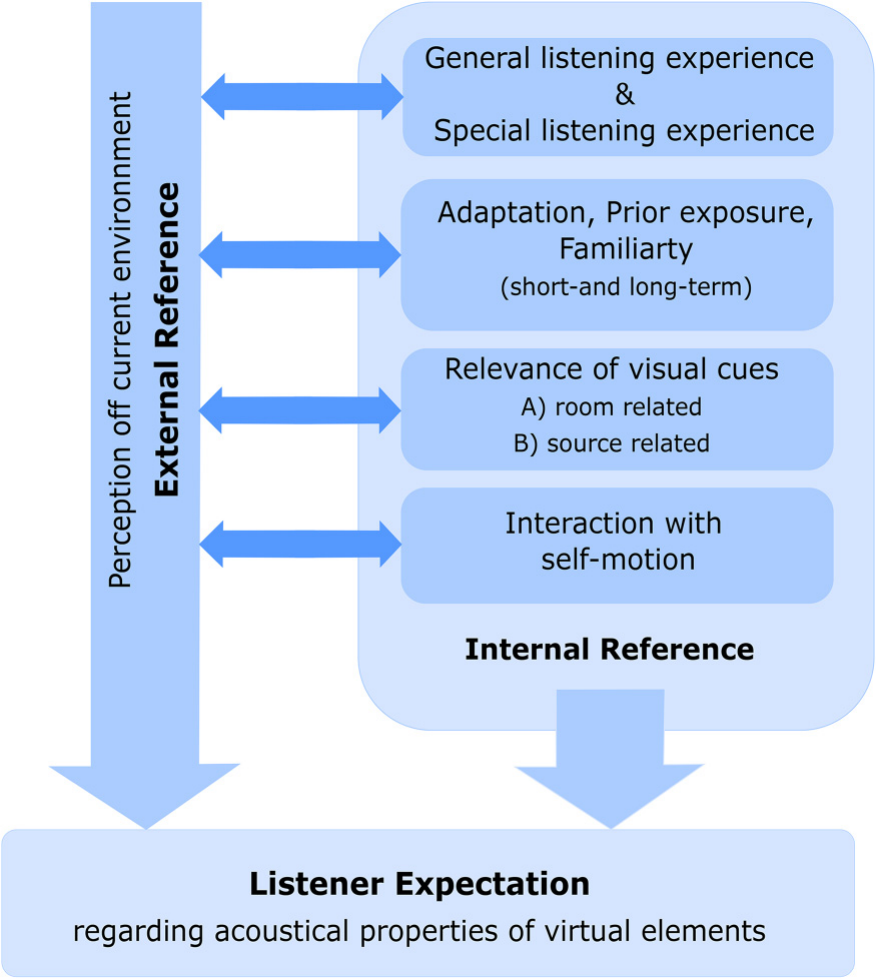
An attempt to outline the role of the different aspects that contribute to the formation of what a listener expects from the room acoustics and the apparent acoustical properties of the sound source in an AAR scenario.

In this section, we propose a concept on the formation of the listener’s expectations with regard to a virtual sound object in an AAR scenario that we developed based on the literature. It gives an overview of the contributing psychological and psychophysical factors that we could extract from the literature so far. The overview may not be exhaustive. We combined identified factors and derived a theoretical concept on their roles and interrelations. Figure 2 visualizes this concept. The role of general experience from everyday listening and the specific experience of expert listeners in their fields have been discussed already. The following subsections discuss the adaptation to room acoustics, the influence of visual information, and cues from self-motion as potential contributors in the formation of listener expectation and present the related state of research.

### a) Adaptation to Rooms - The Effect of Prior or Longer Exposure

People can be experienced listeners not only due to their profession but also with respect to their everyday environments like their living room at home or the office they work in. Whether a listener knows a room or has been exposed to the acoustics of a given room for at least a certain amount of time has an effect on the perception of the reflections and arising expectations regarding the properties of the reproduced room. The results of several studies indicate that previous exposure to a room influences the perception of its acoustics. Adaption effects have repeatedly been reported in the context of speech intelligibility, and echo suppression (Zahorik, 2011; Clifton et al., 1994).

The law of the first wavefront describes the effect that the localization is dominated by the direction of the direct sound, even though early reflections arrive from other directions within a short time after the direct sound. If reflections arrive later or exceed a certain energy level, they start to be perceived as a separate sound event. This is described by the echo threshold. The echo threshold is not a fixed set of values but varies with various physical parameters, the type of signal, and the context. When listening to a specific early reflection pattern for some time, the echo threshold rises, while a sudden change of the pattern decreases the echo threshold significantly. Clifton explains this with expectations arising from adapting to the spatio-temporal pattern and a violation of these expectations by sudden changes (Clifton et al., 1994). She summarizes that “these expectations are most likely based on the listeners’ accumulated experience in highly variable acoustic environments […]” (Clifton et al., 1994, p. 1526). In real acoustic scenes, these changes would relate to sudden movements or a change of the room (acoustics). Keen & Freyman (2009) hypothesizes that listeners form a model when they experience a sound in their surroundings. This model is quickly discarded once the acoustic environment changes. Seeber & Clapp (2020) refer to this process as adaptation. When the listener is able to gain a deeper understanding of the room, for example, by walking around and exploring the room acoustically, an abstraction of the room may occur. This could manifest itself as improvement in speech intelligibility or other complex tasks. Experiments by Seeber et al. (2016) show that previous exposure to the room increases source localization accuracy, and the improvements due to one position in the room also hold for other positions and directions in the same room. Motion may help to speed up the process of understanding the scene, like estimating the size and geometry of the room and the properties of the sound sources (Brandenburg et al., 2020).

Shinn-Cunningham (2000) discussed a similar effect with regard to distance perception. DRR is an essential cue for distance perception. However, in different rooms, equal DRR values correspond to different distances. Thus, the human auditory perception needs to adapt the interpretation of the acoustic cues. Motion is likely to be helpful with this as well. Klein et al. (2017b) showed that a previous short training to the acoustics of a room influences the evaluation of externalization. This can reduce the perceived room acoustic mismatch (room divergence) in a binaural reproduction when only virtual sound sources are audible. However, it remains open whether this training effect is still observable in augmented reality scenarios, where a real sound source is usually present throughout the AR reproduction in the given room.

In general, the adaptation process is not yet well-understood. Some experiments show high inter-individual differences in the learning process (Klein et al., 2017b). The relevant time intervals are unknown, too. In experiments with simple click intervals, adaption effects can be measured after a few hundred milliseconds (depending on the number of training clicks). For reflection suppression to increase the speech intelligibility, Zahorik (Zahorik, 2019) mentions a duration of about one second while experiments with effects on externalization report adaptation over several minutes (Klein et al., 2019).

Augmented reality could be a special case regarding the adaptation to room acoustics. In an AR application, listeners can compare the acoustics of virtual sound sources to the real ones. This side-by-side comparison makes it easy for the listener to discover differences. These ambiguities in the acoustic cues could slow down or prevent adaptation processes altogether.

In summary, the question is how these aspects should be addressed in an AAR reproduction system. Evaluation methods should address this phenomenon by preventing adaptation at all or by incorporating a well-defined amount of training. Further research is needed to define the factors contributing to or preventing adaptation.

### b) Relevance of visual cues

Does the visual impression of a room raise expectations about how the room sounds? In addition, the sound source should be considered and whether it is represented by a real visible or a virtual visual object or whether there is no corresponding visible object at all. It is more convenient to discuss the influence of visual cues by roughly dividing them into two different groups - room-related visual cues and source-related visual cues.

Room-related visual cues

Seeing a church but hearing a small dry room, even non-experts will notice that this does not match. But which role does the expectation rising from a visual room impression play in the perception of slight mismatches in room acoustics? This question is also of great interest for virtual environments, where suitable room acoustic impressions need to be created for fictive, modeled visual rooms (Remaggi et al., 2019).

Udesen et al. (2015) observed that being in a different room affects the perception of binaural reproduction over headphones in terms of externalization. The authors conclude that the visual impression of the other room caused this effect. However, the perception of the surrounding room is multi-modal. Entering a room, hearing the own footsteps, or having a conversation in the room will contribute to the overall impression of the room and consequently to the expectations a listener develops with respect to the reproduced room. The experiment does not allow to draw conclusions about the effect of the visual room impression.

Gil-Carvajal et al. (2016) showed that a mismatch of virtual and real room affects distance perception. The participants were divided into two groups which were provided either with visual or auditory information. A modification of the visual room information did not affect any of the investigated attributes. In contrast, if the real room was more reverberant than the virtual room, the perceived auditory distance decreased significantly. Werner et al. (2016) observed increased externalization for the case that the room with dummy loudspeakers was visible compared to listening in the dark. This effect occurred for all tested combinations of visual and auditory rooms. It has to be noted that this study was conducted without the consideration of head motion which is known to affect externalization. A study investigating the same question with interactive listener motion would be of interest.

In a study by Schutte et al. (2019) the presence of a visual representation of a room did not affect the ratings of reverberance (They asked in German for ’Wahrgenommene Verhalltheit’). In addition, no significant difference was found for a visual presentation of a different room compared to the convergent audio-visual room combination. However, this study considered only three rooms that differed substantially in their reverberation time (bedroom with RT=0.3 s, office with RT=1.5 s, factory with RT=3.4 s). Such obvious deviations in reverberation are likely to overshadow potential smaller effects due to the visual impression. The same research question should be investigated with a larger set of rooms and smaller differences in the reverberation time or reverberance.

Another interesting case is the electroacoustic extension of reverberation, providing control over the reverberation time RT. Such systems change the acoustical properties of a room considerably, but in a well-tuned setup, the reverberation can sound very natural, and often listeners do not notice that they do not hear the room they see, for example Doire et al. (2016).

With regard to the early reflections, Bishop et al. (2011) showed that visual stimuli from a light-emitting diode arriving from the same direction and distance as the direct sound (lead) or a reflection (lag) could affect the echo suppression. The authors explain that with the availability of cross-modal evidence of an object’s existence and location in space. “This interaction is robust to short-term learning effects and critically depends on audio-visual temporal alignment” (Bishop et al., 2011, p. 223). However, this experiment is limited in its ecological validity regarding the role of the visual room impression on the listener’s expectation of the auditory room impression.

In a study by Klein et al. (2017a) listeners had the task to assign acoustic representations to the visual perspective at different positions in a room. A dynamic binaural auralization based on measurements in a real room in conjunction with 360° visuals has been used. Without prior knowledge about the acoustics of the room, listeners were not able to solve this task. After audio-visual training, half of the participants learned to assign acoustic and visual representations correctly. The exploration behavior can explain individual differences in learning success during the training phase.

In summary, it can be said that the influence of visual room impressions on the expectations a listener has towards the room acoustics is not yet well understood. First of all, the mentioned drawbacks of the listed experiments indicate that developing a suitable method to investigate the influence of a visual room impression is challenging. The results of the few studies conducted so far suggest that this influence is limited. Instead, the (first) acoustic impression seems to be more critical for the room matching process.

Source-related visual cues

Source-related visual cues are a very different case. If a visual object represents the sound source, an adequate matching of the virtual sound source in terms of localization, source extension, and source directivity is necessary. A slight audio-visual mismatch in localization may already reveal the virtual object or even break down the auditory illusion (Neidhardt & Zerlik, 2021; Pike et al., 2014). For example, non-individualized binaural reproduction can lead to slight localization errors and coloration. Headphones can cause similar effects if their influence is not sufficiently corrected. In addition, accurate head tracking and an adequately quick response of the reproduction are essential. However, in reverberant rooms, room acoustics influence the perception and localization of the sound source to a certain extent. Especially early reflections are known to affect the apparent source width (ASW) (Kuttruff, 2017), and the DRR is known to affect auditory distance perception (Zahorik et al., 2005). Regarding the perceived audio-visual coherence of shape and spatial extension of the sound source and its directivity, many open questions remain.

In the challenge of perfectly matching the auditory impression to source-related visual cues, an effect called ventriloquism (VE) plays a role. It describes the illusion that the location of a sound source is perceived at the location of a dominant visual stimulus even though the visual and acoustic stimulus are emanating from different directions. This effect can even persist when the visual stimulus is turned off after a settling-in period which is referred to as the ventriloquism aftereffect (VAE). To take advantage of this effect, three main constraints to an audio-visual match have been described. These include spatial, temporal, and context-related constraints. Throughout the years, several studies have investigated these effects, primarily for different spatial and temporal discrepancies. Results show that visual information has a dominant role and can shift the perceived sound source location. Small spatial differences up to $15^{\circ }$ in azimuth or elevation result in a fused audio-visual localization (Slutsky & Recanzone, 2001). For larger spatial deviations between the visual and acoustic stimulus, for example up to $20^{\circ }$ in azimuth, the perceived location of the sound source is significantly shifted towards the visual stimulus (Bertelson & Radeau, 1981; Wozny & Shams, 2011). The visual distance to an object representing the sound source also affects the auditory distance perception and dominates the audio-visual distance judgment (Medonça et al., 2016). A more recent study by Hládek et al. (2020) investigated the VE and VAE for auditory distances between 0.7 and 2.03 m for fixed 30% relative shifts of the visual component. The study was conducted in a small semi-reverberant room with 183 participants. It was found that the VE was constant on a logarithmic scale at 72% of the visual displacement. This indicates that this effect is independent of the distance and whether the fixed shift was closer or farther. In contrast, the VAE showed a dependency on the stimulus direction with a maximum of 44% of the visual displacement when the visual stimulus was placed farther away and 31% for a placement closer to the listener. Moreover, their findings indicate that different neural processes are responsible for the VE and VAE.

Although the visual cue mostly dominates the perceived location of an audio-visual stimulus, it is also possible for the VE to occur vice versa. Alais & Burr (2004) showed that if the localizability of a visual stimulus is badly degraded due to blurring, the sound stimulus takes over the dominant role. They conclude that the VE is a result of a bi-modal integration where the visual and auditory spatial cues are weighted according to their noisiness. Several models have been proposed to describe this audio-visual interaction. A more detailed summary on this topic are provided by Medonça (2020). Stenzel et al. (2019) propose to evaluate audio-visual fusion using reaction time measurements to reduce the variances between studies compared to evaluations using continuous scales.

Postma & Katz (2017) investigated the influence of visual distance to the sound source object on ASW, listener envelopment (LEV), and other attributes in a virtual concert hall presented in a three-walled CAVE system. A non-individualized headphone-based binaural reproduction was used for the audio presentation. ASW and LEV ratings were not significantly affected by a visual position mismatch. A study on the perceptual matching of ASW with respect to the visible dimensions of a source object could not be found, although this is interesting with a scope on the case of small rooms. ASW of the same sound source can vary significantly among different positions within a small room (Schneiderwind & Neidhardt, 2019) and even more between different rooms. Another interesting question is whether there is an equivalent effect like ventriloquism for ASW.

In general, there are two opposing effects of source-related visual cues. On the one hand, a visible object representing the sound source can draw the localization of the acoustic object towards that object. On the other hand, the availability of a visual object makes the listener more critical in accepting an auditory illusion as real or at least plausible. Therefore, it has to be investigated in which cases a visual anchor makes a listener more critical or less sensitive to minor inaccuracies in the acoustic properties of a virtual sound source.

### c) Relevance of cues from self-motion

Spatial auditory illusions are compelling if they endure an interactive listener motion in 6DOF. When a listener actively changes his position or (head-) orientation, certain expectations arise for the change of the sound reaching the ears in correspondence to this motion. These expectations are also based on the experiences from everyday listening. For understanding how to satisfy these expectations, it is necessary to understand the role of information from our sense of motion and self-motion in spatial hearing. The vestibular system is one of the contributors to the conscious sensation and guidance of motion and posture. Another contributor is the proprioceptive system. Proprioception refers to the sense of self-motion based on sensory-motor information (Proske & Gandevia, 2012). So far, only selected aspects of their role in the perception of spatial sound have been addressed. Binaural technology has become an essential tool for investigations in this field.

It is known that head motion facilitates sound source localization and improves localization accuracy (Thurlow et al., 1967; Mackensen, 2004; Honda et al., 2013) and helps to resolve front-back ambiguities (Pöntynen & Salminen, 2019). Kim et al. (2013) observed that listeners move their heads over a wider range when judging source width and listener envelopment than for sound source localization. For the evaluation of timbre, the range of head rotation in azimuth was very low compared to the other tasks. For active changes of the elevation angle, the differences were rather small and primarily not significant. Active head rotation is also known to improve externalization in a dynamic binaural reproduction (Brimijoin et al., 2013). Hendrickx et al. (2017) reported that this improvement in externalization persists even after dynamic cues were omitted.

Kondo et al. (2012) investigated the influence of active head motion on auditory scene analysis. When listening to a complex scene, the incoming flow of acoustic information is organized in streams that are not instantaneous but built up over time. The organization of streams can also be reset if sudden changes occur in the scene. When moving the head in a stationary scene, the acoustic stream changes as well, but the listener should understand that the scene did not change. Kondo et al. (2012) observed that with the onset of the motion, the organization of streams was partially reset and reorganized under the consideration of the spatial cues provided by the movement.

Wallmeier & Wiegrebe (2014) showed that vestibular and proprioceptional information provides helpful cues for the human echo-acoustic orientation. The observation may be based on the same mechanisms reported by Kondo et al. (2012).

Active self-motion and exploration behavior give access to different cues that the auditory system could use. On the one hand, a listener gains additional information from positional disparity. On the other hand, dynamic auditory cues about the current change of the sound reaching the ears are available.

Listening to a scene subsequently from different positions and perspectives provides more spatial information about it. The brain can collect this information and interpret it. Generally, humans can create a cognitive map of their environment (Epstein et al., 2017). Maybe this is possible based on the gathered auditory information. This is not only the case when walking through the whole scene, but this starts already with little head motion for source localization. Especially for sound sources in the front, without movement, often in-head localization occurs. Turning the head provides additional information, and when turning it back to the original position, the same ear signals are interpreted differently considering the collected information.

On the other hand, during motion, the auditory system can use dynamic cues like the current change of the sound level or the current change of DRR. For example, the acoustic τ (time-to-contact) addresses the current change of sound intensity during a motion of the listener, or the sound source (Shawn et al., 1991; Guski, 1992). If the listener moves, dynamic auditory cues are available that can be analyzed in combination with proprioceptional cues related to the own motion. In this case, the listener will expect that a certain motion is connected to a certain change of the sound field. This may also be a result of long-term listening experiences. However, this is still more of a hypothesis. Studies in this field a rare.

For AAR, it is important to be aware that listeners notice if the simulated sound field does not change according to their motion. For changes in the head orientation, very early room reflections can cause an image shift effect that influences the apparent source location. In addition, system latency, an insufficient angular resolution of the dynamic reproduction, and a lack of individualization are the primary source for erroneous localization in AAR. For 6DOF motion, the listener expects an adequate relative change of the auditory distance perception, which is also influenced by the acoustics of a room. Listeners notice, for example, if the sound source moves along while moving away from the sound source (Neidhardt et al., 2018).

Furthermore, exploring the room acoustics by active listening with self-motion likely facilitates the process of adaptation to a room or abstraction, as discussed in the previous section. More details of the role of the listener’s active self-motion in the creation of auditory illusions are discussed by Brandenburg et al. (2020).

### d) Interpretation and Understanding of room acoustical cues

Some listeners draw special information from room acoustical cues, for example, for the detection of an obstacle or a reflector. Commonly, this requires a certain minimum change in the perceived sound that the listener can recognize. For example, in an experiment by Neidhardt et al. (2017) virtual acoustic walls were created with dynamic binaural headphone reproduction. These walls were not visible, but their acoustic effect was integrated with the auralization. The participants could control a speaking avatar with their motion and basically listened with the avatar’s ears. The direction of the virtual wall was randomly changed for each trial, and the task was to determine the direction of the closest wall. The participants had to turn themselves until they thought the avatar is facing the wall. This procedure was repeated for different distances to the wall in different virtual rooms. In close distances, most listeners were able to localize the direction of the wall quite accurately. At a distance of two meters, most participants reported that they perceived a change in the reverberation during their self-rotation but found it hard to tell which of the changes is an indicator for the wall. This is a slightly abstract example. However, it highlights the fact that adjusting only selected room acoustic parameters, like the reverberation time, may not be sufficient in providing the acoustics cues, for example for estimating the directions of the closest wall, some other reflector, or generally the geometry of the room or the environment.

This opens an own field of research, which deals with the interaction between physical detail and contextual information on the interpretation of the scene or environment. Another interesting question arises, for example, if the user of an AAR system hears a voice from further away. Is a person speaking or is a loudspeaker reproducing the voice? If a person is speaking, is this person talking to us as the listener in this scenario? How well can listeners extract such information from real acoustic environments? Do listeners use such physical details for interpretation or do they rely more on contextual information? Which room related cues are used? Does simplification affect them? Does the cocktail party effect still have a similar impact as in real sound fields if the reverberation of several sound sources was created, for example with wrong or simplified early reflection patterns or maybe with the same late reverberation tail?

Room acoustics are not only perceived in terms of reverberance. Room acoustics also subconsciously influence how we perceive sound sources and how we extract other information from our acoustical environment. The concept of perceptual room matching assumes that the listener expects that a room ’behaves’ as it does in reality. One exception may be the desired creation of an unnatural room, for example, for artistic reasons. Then the listener probably wants the unnaturalness to be obvious. However, this case is not within the scope of this review.

## Required Accuracy of Physical Properties

The previous section discussed the formation of a listener’s expectation. In this section, we assume the listener now has these more or less specific expectations, for example, regarding the position of the sound source, its sound level, the width of the source, or the reverberation of the room. The required physical accuracy of the auralized room acoustics is determined by the range of variation in room acoustics that still fulfills the listener’s expectations. JNDs denote the minimum change of a physical parameter that causes a change in perception. However, a slightly noticeable change in room acoustics does not necessarily lead to a perceptual mismatch with respect to the expectation.

The parameters that can be tuned or adjusted to achieve perceptual room matching usually depend on the implementation and the algorithms applied for the binaural synthesis. However, all synthesis methods and reproduction approaches have to meet the perceptual requirements.

Determining the most critical parameters becomes more difficult because we have to assume a nonlinear combination of parameters when realizing plausible reproduction. Isolated evaluation of specific parameters might be misleading, and the importance of certain parameters could be underestimated.

Additionally, the purpose and capabilities of the application can shift the weight of the parameters. For example, in 6DOF applications dynamic cues are very important for externalization and can overrule the importance of other parameters. But in applications without tracking of pose and position, these cues are not available and therefore other parameters gain in importance.

**Figure 3.**
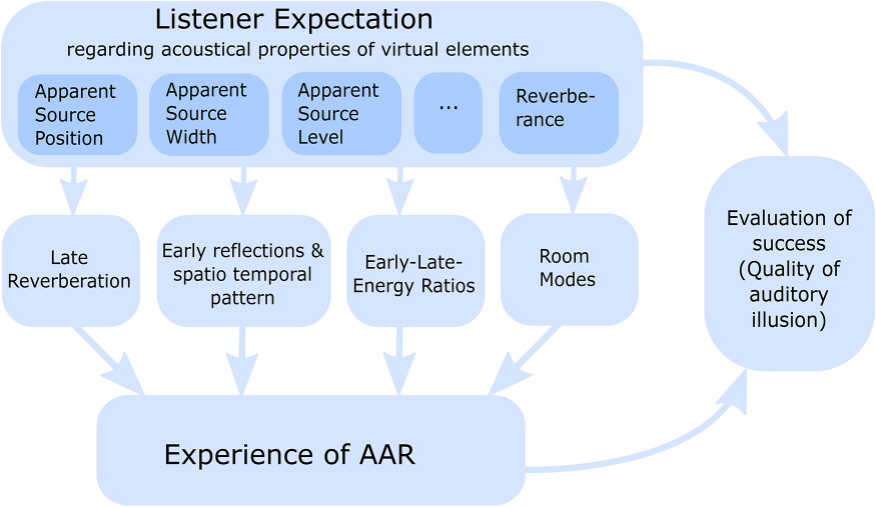
The listener has certain expectations regarding the acoustical properties of the sound source and the room. This section discusses, how limitations of the physical accuracy affect the inducement of convincing auditory illusions in AAR. The discussion is structured by the segments of a (binaural) room impulse response as it is visualized. The evaluation of the success of perceptual room matching is topic of the subsequent section.

Thus, the following section focuses on general room acoustic properties, but also discusses selected tuning parameters of existing implementations. The discussion of the required physical accuracy to create convincing auditory illusions in AAR is structured by the different parts of a room’s acoustic response. This structure is visualized in Figure 3. Subsequently, the properties of small rooms’ late reverberation, their early reflections and the corresponding spatio-temporal, the early-to-late-energy ratios, and occurring room modes are analyzed. Which of each component’s properties have to be modeled with which accuracy or level of detail to satisfy the listener’s expectations regarding the auditory appearance of the sound source and the room?

That is the key question for the development of efficient AAR systems. This section will discuss this question for each of the components step by step.

### a) Late Reverberation

Theoretical considerations assume that a certain time after switching on the sound source in a room, a diffuse sound field is established. According to its definition, a diffuse sound field has a uniform sound pressure distribution and a uniform distribution of incident sound intensity. Perfectly diffuse reverberation is hardly achieved in real rooms. Romblom et al. (2016, p. 1) claim that directional components in non-ideal diffuse field reverberation “may be a previously unrecognized component of spatial impression.” However, starting from a certain point of time after room excitation, the listener cannot perceive direction-dependent differences. This may even hold for different positions if room modes remain at a negligible level.

This point of time is referred to as the perceptual mixing time (Lindau et al., 2012). It can be used to simplify the synthesis of the late reverberation by keeping the late part of the impulse responses constant for the different directions and possibly also for the position. Very few studies considered positional changes in the determination of the perceptual mixing time were conducted (Meesawat & Hammershøi, 2003) and Neidhardt (2021) even with interactive walking. Pörschmann & Zebisch (2012, p. 544) picked measurement positions “in the diffuse field of the sound source”. While Lindau et al. (2012) chose to place the speaker at twice the critical distance in the corresponding room, Meesawat & Hammershøi (2003) placed them 1.5 m from the listener. This results in strong direct sound and a high DRR compared to other positions in the room. This is not representative of 6DOF. Neidhardt (2021) created a test case with low direct sound energy by turning a directional sound source away from the listener. Typically, values between 30 and 60 ms were found for the perceptual mixing time in small rooms (Lindau et al., 2012; Meesawat & Hammershøi, 2003; Pörschmann & Zebisch, 2012; Neidhardt, 2022). Especially for 6DOF scenarios, an in-depth investigation of the perceptual mixing-time that considers the occurrence of room modes is still pending.

A certain time after the beginning of the excitation of the room, often referred to as the physical mixing-time, the reverberation can be described by a statistical time-frequency model. Such models commonly include parameters describing the frequency-dependent exponential decay as well as gaussian statistics of the reverberation after about 30–50 ms (Traer & McDermott, 2016). Examples are the spectral energy decay curves and interaural cross-correlations. Several methods have been proposed to synthesize the late reverberation tail based on the information given by an omni-directionally measured RIR. One approach is to extract the energy decay relief (EDR) and frequency-dependent decay curves. These can be scaled according to the reverberation properties of the desired room (Jot & Lee, 2016) or by extracting envelopes of subbands resulting from a filterbank analysis and applying them to shape a binaural noise sequence (Pörschmann & Zebisch, 2012; Arend et al., 2021).

Another approach is the use of feedback-delay networks (FDNs). Based on a direction-dependent target reverberation time, Alary et al. (2019) create directional anisotropic reverberation with a directional FDN. Depending on the specific application, the trade-off between spatial accuracy and computational costs has to be considered. A perceptual evaluation remains to be conducted.

Regardless of whether the late reverberation is simulated or measured and adjusted, the same challenges apply to room matching. In both cases, the relevant parameters have to be known to create convincing synthetic reverb or modify recorded reverb. While simulated reverberation offers more flexibility for changing certain properties, it also requires more effort to create natural-sounding reverb in the first place.

Djordjević et al. (2020) conducted a MUSHRA listening experiment comparing the perceived naturalness of five different reverberation algorithms, including an FDN and a scattered delay network (SDN) method. SDNs, in contrast to FDNs, render the direct path component as well as the first order reflections in accordance to a room model (De Sena et al., 2011). The experiment did not consider listener motion (Djordjević et al., 2020). The results suggest that SDNs create a more natural-sounding reverberation than FDNs. However, the researchers considered only one specific method of FDN implementation. The underlying test method does not verify if the quality requirements for a high-quality AR reproduction are met. No results of synthetic late reverberation evaluated in an augmented reality test scenario could be found for this review. The general impression of the authors is that currently available implementations are quite successful in adjusting the late reverberation in accordance with a given room. However, studies evaluating the suitability for AR scenarios in-depth are still pending. One challenge is the development of appropriate evaluation methods.

Reverberance can be predicted quite reliably by the parameter *perceived reverberation (pRev)* based on binaural auditory models (van Dorp Schuitman et al., 2013; Vecchi et al., 2017). This approach can be applied directly to the audio stream without the need to extract the BRIR. The results show that reverberance correlates well with the EDT. However, signal properties like the level and the spectral content have a strong influence on pRev. The results show good alignment of the model with listening test results.

Furthermore, IACC ${}_{Late}$ is associated with the perceived listener envelopment (LEV) in concert halls. It usually considers the reverberation starting from 80 ms after the direct sound. Soulodre (2004) as well as Beranek (2010) propose to calculate a physical measure for LEV summing a level component and a spatial component which is determined based on the IACC ${}_{Late}$ . LEV has not been investigated in the context of AAR in small rooms or with regard to 6DOF. A stably low interaural coherence can also be an indicator of the diffuseness of late reverberation and the mixing time. In a coherence-based estimation of the mixing time, the moving short-time interaural cross-correlation STIACC can be helpful. However, Alary et al. (2021) analyzed spatial room impulse responses (SIR) recorded with a 32-channel spherical microphone array. They determined a mixing time defined by a stable minimum of coherence and showed that after this mixing time, still directional components can be found in the reverberation of the considered concert halls. These components are audible. An investigation of the same question in small rooms would be of interest. It also remains open whether these components play a role in perceptual room matching.

### b) Early Reflections, their spatio-temporal Structure and Relation to Direct Sound

One of the major questions in the field of creating perceptually matching room acoustics is the role of early reflections and the sensitivity to deviations in their spatio-temporal pattern, as well as the properties of single reflections.

The latest in-depth evaluation of room acoustical simulation tools (Brinkmann et al., 2019) revealed that the perceptual difference between measurement and simulation are deviations in apparent source position and coloration. According to the authors, these differences can largely be “traced back to the simplified use of random incidence absorption and scattering coefficients and shortcomings in the simulation of early reflections due to the missing or insufficient modeling of diffraction” (Brinkmann et al., 2019, p. 1).

Also, in the interpolation or extrapolation of BRIRs, considering all the details of the early reflection pattern is challenging. Therefore often simplifications are applied (Bruschi et al., 2020; Brinkmann et al., 2020; Müller & Zotter, 2020). For this reason, it is crucial to understand which level of detail is required to provide a room acoustic impression without perceptual discrepancies. This is especially interesting for sources and listeners moving in 6DOF because the relative spatio-temporal pattern changes with each position change.

Adding one single strong reflection to the direct sound will cause a comb filter effect, which can lead to audible coloration (Bech, 1995, 1996; Brunner et al., 2007). The character of this effect changes with the delay of the reflection and its individual properties with regard to the direct sound. If an AAR system aims to create authentic auditory illusions, such effects have to be considered. In these cases, an estimation of the geometrical arrangement of the surroundings is necessary. However, even with the goal to create a virtual copy of a real sound object, it is not clear whether listeners precisely expect the original progress of timbre during motion.

It is known that early reflections arriving within 1–7 ms after the direct sound can cause a shift of the apparent source position by an effect called summing localization. For reflections arriving after that time range, the localization of the sound source is determined by the spatial cues of the first arriving wave-front (Wallach et al., 1949). This so-called *precedence effect* has been studied intensely since its discovery. Litovsky et al. (1999) provide a detailed review of investigations until 1999. Brown et al. (2015) reviewed additional results of the following 15 years. Commonly, the experiments are based on a lead signal and a delayed and, in many cases, attenuated copy of it, the lag. This test paradigm is a keen simplification of sound propagation in rooms. Brown et al. conclude that “a more ecological understanding of the precedence effect as a mechanism for the preservation of accurate sound localization in reverberant environments […] will ultimately require more ecological approaches to its study” (Brown et al., 2015, p. 24). Few studies considering more than one lag, more than one lag direction, or signals different from click-trains have been addressed in this review. Moreover, representing a reflection as an ideal impulse is a substantial simplification either. Natural reflections usually underlie a spatial and temporal spread that depends on the directivity of the sound source, on the reflection properties of the corresponding surface, and the geometrical constellation of sound, reflector, and receiver. It was shown that these natural reflection properties result in a considerably different appearance of the precedence effect (Robinson et al., 2013; Wendt & Höldrich, 2021) and for surfaces in close distances (< 50 cm) additional near-field effects occur (Paasonen et al., 2017). Adding first-order image source reflections to a rotating directional sound source in a small room can lead to considerable shifts of the apparent source direction (Zotter & Frank, 2015). The addition of further reflections did not cause additional localization shifts, only smoothed the transition. Similar observations were obtained by Steffens et al. (2021). Early reflections also influence other spatial aspects than the apparent location of the sound source (Bech, 1998). One example is the apparent source width (ASW) (Barron & Marshall, 1981) and the apparent sound level of the sound source. The apparent sound level is probably not critical for audio-visual coherence. An interesting question is whether this increase of the apparent sound level can reach the threshold at which it becomes critical for perceptual room matching. For ASW IACC ${}_{early}$ is considered a good indicator (Okano et al., 1998). However, IACC ${}_{early}$ varies considerably with the orientation of the head in relation to the sound source and can also vary with distance. Thus, maybe a matching of IACC ${}_{early}$ is only interesting for the person facing the sound source. In addition, ASW varies with the reflection’s angle of incidence (Johnson & Lee, 2019). More studies considering the diverse properties of natural room environments are required.

Shinn-Cunningham & Ram (2003) observed that the sensitivity to differences in the early reflection pattern due to different positions in the room is limited, and so is the understanding of the own listening position in the room. Studies with blind people (Kolarik et al., 2017) reveal that long-term training can improve the capability of extracting information about the environment from auditory impressions. Klein et al. (2017a) found that after a short training, only very few listeners could confidently assign the listening perspective to the corresponding visual perspective of the room if the direct sound is kept constant. Only special cases, like a listening position close to a wall, were recognized reliably by most participants. In the case of a weak direct sound, for example, behind a sound source, the audible differences are most prominent, as a considerable shift of the apparent source location towards the first dominant reflection (Schneiderwind & Neidhardt, 2019).

For an interactive approaching motion towards a virtual loudspeaker in two different rooms, the plausibility remained unaffected by keeping the spatio-temporal pattern of the early reflections constant over the given distance of 2 m (Neidhardt et al., 2018; Neidhardt & Kamandi, 2022). In these experiments, the translation line was located in front of the virtual loudspeaker. Minor differences in the reverberant part could be masked by the strong direct sound as discussed, for example, by Buchholz et al. (2001) and Welti & Jensen (2003). If the loudspeaker was turned by $180^{\circ }$ , facing away from the listener, the plausibility was seriously affected, if the same approach was used (Neidhardt, 2021). This confirms the findings by Zotter & Frank (2015); Steffens et al. (2021) and shows that auralizations created by the various signal processing approaches should be tested with such an indirect reproduction scenario because it is much more critical than the listening positions in front of a sound source.

A perception-based simplification algorithm was introduced by Hacıhabiboğlu & Murtagh (2007) which aims at reducing the number of early reflections needed for the auralization. Based on a prediction model, only image sources that contribute to the perception of the sound field are selected for the auralization. In the perceptual evaluation, the proposed method showed no significant degradation concerning localization performance and perceived spatial quality features such as Presence, Spaciousness, and Envelopment.

There are further details in the structure of early reflections that have received only little scientific attention so far. For example, considering edge diffraction in the simulation of early reflections has been shown to be audible for selected signals in an ABX test paradigm with monaural auralizations (Torres, 2001; Calamia, 2009). In the latest round robin comparison showed that room simulations without a consideration of edge diffraction still produce plausible auralizations (Brinkmann et al., 2019). In AR-scenarios, pychoacoustic evaluation of such details in the early reflections like edge diffraction, but also near-field and shadowing effects are still pending.

In summary, it is known that the sensitivity to the physical details in the early reflections and their spatio-temporal pattern of arrival at the listener is limited. However, simplifications in the early reflections can cause noticeable coloration, change the apparent source width and affect correct source localization in direction and distance, which can also affect audio-visual coherence.

Studying the effect of the various physical parameters in the spatio-temporal pattern of the early reflections on perception requires to consider its interrelation with the properties of the direct sound as well as the late reverberation. In addition, the visual impression and the listener’s expectation have to be taken into account. The perception of early reflections is a complex field which is not yet understood in detail. This section can only give a rough insight into the multiple facets and point out that still more research is necessary to acquire a full understanding.

### c) Early-to-Late-Energy-Ratios and their relative change

Early-to-Late-Energy-Ratios (ELR) play an important role in predicting the perceptual quality of concert halls. Examples are the clarity indices C80 and C50 and the direct-to-reverberant-energy-ratio DRR as special cases of ELR. The DRR is known to be an essential cue for the auditory distance perception in rooms and is known to vary with the room (Zahorik et al., 2005). Therefore, the DRR is a relevant parameter for perceptual room matching.

Neidhardt & Kamandi (2022) observed that the plausibility of walking towards and away from a virtual sound source was affected if the relative change of the DRR did not reflect the actual change in the reproduction room. When reproducing the BRIRs measured in the much drier listening laboratory in a seminar room, the sound source moved along and the change of the distance did not match the own motion in the room. The perceived change in distance was not sufficient. The relative change of the DRR did not match the room. Similar observations were made by Neidhardt & Schneiderwind (2021).

Wendt et al. (2017) and Laitinen (2015) showed that the variation of directivity influences distance perception. A sound source with a different directivity or orientation will impact the position-dependent progress of the direct sound energy and consequently will affect the progress of the DRR. It remains open how accurately the progress of the DRR has to be imitated to achieve plausibility. This could also be a matter of the spatial, temporal, and spectral distribution of the direct and the reverberant sound energy.

In concert halls, C80 is used to estimate the perceived clarity of the room acoustic with (orchestral) music, while C50 is associated with speech (Kuttruff, 2017). Both parameters are interesting because they consider the perceptual fusion effects between direct sound and early reflections that occur in rooms based on the mechanisms of the precedence effect. Furthermore, they address that the time range of this perceptual fusion depends on the type of source signal. For small rooms, the clarity indices are also interesting due to their correspondence to the perceptual mechanisms like temporal integration of early reflections. For strong direct sound, there is a strong correlation of C80 and C50 with the DRR. However, generally, the clarity indices are less sensitive to variations of the direct sound energy at the listener’s position in 6DOF, especially for sound sources with a pronounced directivity. C80 was mentioned to correlate with auditory room size and distance perception (Cabrera, 2007a). For speech signals, C50 may be even better. From a theoretical point of view, clarity indices are also interesting to estimate the perceived distance for cases of lower direct sound. They also mirror the auditory horizon effect in auditory distance perception. Accurate distance perception and its relative change with listener motion are essential in the creation of 6DOF systems, in particular for audio-visual coherence.

Only very few studies address the estimation of JNDs for ELRs, for example, Larsen et al. (2008) investigated the JND of DRR. However, such JNDs are likely to vary with the temporal, spatial, and spectral distribution of the energy, which has not been considered in JND estimation so far. In addition, there are still debates about the criteria to estimate a suitable transition time (range) between the early and late part of the reverberation.

Generally, in the case of motion in 6DOF, there is always a relative change of the discussed parameters. This change depends on the room and the movement. This raises the question, whether the change is perceived as characteristic for a given room.

### d) Consideration of room modes

According to Knudsen (1932, p. 36) “the qualities of all sounds, such as speech and music, are changed by the resonant properties of rooms. This change may be of a large magnitude in small rooms. Thus, certain low-frequency components which agree with natural frequencies of a room may be intensified as much as 20 to 25 dB.” Knudsen also points out that the effect is especially strong for wave-lengths in the room dimensions range. The transition between the low frequencies, which are dominated by separate room modes, and the high frequencies that exhibit a dense modal overlap with Gaussian properties is smooth and continuous. Therefore, a limiting frequency can hardly be defined. Schroeder & Kuttruff (1962); Schroeder (1996) proposed a 3-fold modal overlap. This resulted in the definition of the well-known Schroeder frequency, which depends on the reverberation time and the room volume. It is one specific frequency value marking a region of transition. Skålevik (2011) argues that the Schroeder Frequency has been “designed and tested as a low limit ensuring the validity of high frequency theory.” Consequently, the value is sufficiently high, but it could be higher than necessary.

Investigations on the perception of room modes have mainly been motivated by the goal to control the modal decay for room acoustic treatment applications (Karjalainen et al., 2004), optimal loudspeaker placement (Bech, 1994; Olive et al., 1994) or the general audibility of spectral irregularities (Bücklein, 1981).

One of the recent studies concerned with the determination of perceptual thresholds of room modes was conducted by Fazenda et al. (2015). The study investigates two different perceptual thresholds as a function of modal decay. The first set of test stimuli consisted of windowed sine bursts representing the excitation of single resonances to determine absolute thresholds. The second set included music signals considering the more complex nature of real signals in terms of temporal and tonal characteristics as they are likely to introduce different masking effects. As expected, the measured thresholds for the musical signals exhibit higher values, therefore lower sensitivity, than for the “single resonance” signals. Generally, the threshold can vary enormously with the type of signal and decreases with increasing frequency. According to the authors, there is still a lack of studies to create a coherent auditory model.

In the context of AAR, room modes have barely been taken into account. Many questions remain open, for example, a sufficient understanding of the listener expectation with respect to room modes is necessary. The listener may prefer a simplified version of the sound field without considering room modes since, in many real rooms, the goal is to suppress them. Room modes are issue for 6DOF listening scenarios, as the listener can walk through the room, and room modes can cause position-dependent fluctuations in the low frequencies. Furthermore, the perception of room modes depends not only on the listener position but also on the source position and sound source directivity. Including these variations in the auralization would require a more sophisticated rendering of room acoustics. An understanding of the perception of modal structures in small rooms will help to optimize rendering algorithms.

One specific case is the determination of the mixing time to simplify late reverberation synthesis for position-dependent reproduction. Lindau et al. (2012) suggested that the occurrence of audible room modes limits the extension of the mixing time concept to position-dependent reproduction. This presumably applies only to frequencies (clearly) below the Schroeder frequency.

Moreover, room modes can impose a practical issue for algorithms based on a sparse positional sampling of the acoustics in a room. Either positions with strong modal effects should be avoided, or the algorithms for post-processing should be robust to their influences.

## Evaluating the Success of Perceptual Room Matching

Investigating, evaluating, and hopefully confirming the successful matching of a virtual (synthesized) room to a real one requires suitable test methods. An obvious mismatch of rooms may be addressed by asking participants whether they perceive the reproduced room as matching for the given environment. If the listener perceives a mismatch, it may be helpful to ask for a free description of the perceived differences to identify potential for targeted improvement.

However, the better the systems get, the less obvious the differences will be. Listeners may not be able to tell that there is a mismatch in rooms, but only that “something” is not right. Consequently, different test approaches are necessary.

For perfect realizations, the goal will be to create an *authentic* virtual sound source that cannot be distinguished from reality, meaning the real version of the sound source in the real version of the environment. In this case, a direct comparison of the real version and the virtual version is of interest, for example, in an ABX-experiment. Brinkmann et al. (2017) showed that under careful consideration of many technical details, it is possible to achieve this goal - at least for speech. With noise as the test stimulus in the same technical setup, the majority of the participants could still perceive differences.

A direct comparison with a real version of the virtual element will not be possible in most applications. Thus, another test paradigm is of interest for ecological validation. Kuhn-Rahloff (2012) proposed to define plausibility as a measure of agreement of the created auditory illusion with a listener’s internal reference. Lindau & Weinzierl (2012) suggested a method to test plausibility by asking the participants whether they are listening to the real or a virtual version of the sound source in a randomized presentation of either of both. Pike et al. (2014) used this method to show the drawbacks of non-individual BRIRs as most participants could identify the virtual version. Lindau & Weinzierl (2012) showed that with individually measured BRIRs, a plausible dynamic binaural reproduction in the sense of the given test paradigm could be achieved. Both experiments focused only on head rotation.

Neidhardt & Zerlik (2021) showed that including the real version of the sound source as a test stimulus increases the detection rate when identifying the simulation. This suggests that the presence of the real version tunes the internal reference. In addition, for creating virtual sound sources for which a real version does not exist, like a speaking animal or a fantasy creature, a different approach to evaluate plausibility is required.

The realization of auditory augmented reality usually requires a wearable reproduction device, which will also affect the perception of the real sound sources in the scene, as discussed for several open and closed headphones by Satongar et al. (2015) and Schneiderwind et al. (2021). Despite the considerable progress in hear-through solutions (Denk et al., 2018; Gupta et al., 2020) perfectly transparent reproduction devices do not exist (yet) (Schepker et al., 2020). This limits the capabilities of AR systems in general and, thus, the possibilities to investigate perceptual room matching. Currently, the perceptual deviation between the real and the auralized room only needs to be below the audible corruption caused by the presence of the reproduction device.

The methods mentioned above are based on a direct or indirect comparison to a real version of the virtual sound object. These have the disadvantage that shadowing effects caused by the hearing devices also had to be considered in the creation of the virtual content. Neidhardt et al. (2018), for example, conducted an experiment where participants had to rate plausibility without hearing the real version. This approach has the advantage that the audible shadowing effects of the headphone or hearable do not have to be taken into account. Thus, the evaluation may be more critical concerning the perception we have in natural listening conditions. The disadvantage is that participants purely rely on their internal reference, which depends on the listener’s expectation and can be inaccurate or even wrong. In the experiment, selected test conditions were perceived plausible by all participants, while others were rated as implausible by every listener. This method is suitable to measure the agreement with the internal reference. However, it was observed that some participants preferred falsified versions over the measured ones, because they expected more audible change over the tested change of distance than there is in reality. This means that even with physically accurate sound pressure at the eardrums, it is not guaranteed that the listener’s expectations are met.

Authenticity and plausibility are overall criteria or attributes for an evaluation of the overall impression. A general impression results from a combination of contributing factors, for example, localizing a sound source outside of the head, the sharpness and stability of the auditory image of the sound source, and audiovisual congruence if a visual object is representing the sound source. These contributing factors are not of equal importance in each augmented acoustic scenario. The content and the context play a role in weighting as well. For example, when creating a virtual bee, which is not visible but audible, it will fly around and make sound only while flying. Sound source stability or a perfectly accurate perceived source position is less relevant. But overall, the impression still needs to be plausible to convince the listener. In contrast, to raise the illusion of statue talking, localizing the speech at the statue’s mouth and providing source characteristics of a person speaking is vital for creating a plausible auditory illusion. Audio-visual coherence is important in this example. This also includes sound source stability and an adequate approximation of directivity and apparent source extension. Externalization is considered crucial for all types of scenarios. Still, it has to be kept in mind that also with real sound sources, in-head-localization can occur (Toole, 1970).

Moreover, indirect or behavioral test methods can help to identify subconscious effects of insufficient matching of room acoustics. For example, it is of interest whether sound source localization works with the same precision for virtual and real sound sources in AR as for real sound sources in normal listening situations without wearing any reproduction device (Satongar et al., 2015). Another interesting approach is to measure the duration of reaction time for certain tasks (Stenzel et al., 2019) in AAR. It is also interesting whether psychoacoustic effects known for real environments, such as the cocktail party effect, comparably occur in mixed reality scenarios. It is possible that in more complex scenes, the psychoacoustic requirements for single sound sources are lower. This is probably also a matter of attention. Such questions again require additional suitable test methods.

## Conclusion

This article reviews the perceptual matching of the room acoustic properties of virtual contents in Auditory Augmented Reality to the acoustics of the user’s actual environment. Occurring acoustic reflections in a room can become audible in terms of reverberance and changes in the auditory image of the sound source like the apparent sound level, apparent source position, apparent source width, and perceived timbre. For creating auditory illusions over headphones that seamlessly fuse with the user’s real environment, the reflection behavior of the real environment has to be imitated well enough by the virtual reproduction.

Despite several decades of research in the perception of room acoustics, the relation between the physical properties of the sound field and perceptual attributes is not fully understood. Seemingly simple aspects like the auditory perception of room size remain mostly unclear. In addition, cognitive mechanisms influence the interpretation of sensory information. Based on the individual listening experience from everyday life and the impressions of the current environment, listeners form certain expectations of how a specific sound source in this environment should sound.

Different reproduction methods vary in terms of their complexity and ability to obtain physical accuracy and have different system parameters that can be tuned to meet the perceptual requirements. However, the perceptual requirements for a given AAR scenario are independent of the implemented algorithms. This article reviews these perceptual requirements for the auralized room acoustics in AAR systems. The following list summarizes the main conclusions we draw from this review.

- With accurate measurements of individual BRIRs and individual hearing device compensation, it has been shown that an authentic illusion can be achieved which is indistinguishable from reality. Authenticity is the most critical quality demand for spatial auditory illusions.
- A seamless fusion of virtual and real sound sources requires the analysis of room acoustics in real time and an incorporation of this information in the synthesis. These practical constraints currently limit the achievable physical accuracy of analysis and synthesis of the sound field.
- The required accuracy varies with the quality requirement of the individual applications, the environment, the virtual content, the context of use, and the individually formed expectation (which can be idealized or even wrong) for the respective scenarios. Acoustical deviations above the JNDs are acceptable to a certain degree in terms of plausibility. There is also doubt that a physically perfectly accurate reproduction would reliably satisfy the listener’s expectations.
- The usefulness of established acoustical room parameters for efficient AAR realizations in small rooms is still subject to research.
- Perfectly transparent reproduction devices are not (yet) available. This limits the capabilities of AR systems in general and, thus, the possibilities to investigate perceptual room matching.
- There is a lack of established test methods to measure the success of perceptual room matching. Such methods are required to determine specific thresholds. Methods based on direct or indirect comparison with real sound sources face the issue of the aforementioned limited transparency of available hearing devices. Ideas for indirect and behavioral evaluations are currently pursued.
- Dynamic acoustic changes arising from listener and sound source movements serve as cues for the human auditory system. Their role in the perception of acoustic scenes and the required level of detail in virtual imitations are mostly unknown. There seems to be a lack of research.

For specific AAR scenarios, it has been shown that plausibility could already be achieved with quite rough approximations. Knowing the perceptual requirements for plausible auralizations will help determine the minimum requirements for the technical specifications regarding the analysis and synthesis of an AAR system. If the goal is to create an authentic virtual sound source or a virtual twin of a present sound source, many technical details like an accurate compensation of the headphone’s transfer characteristics and the impact of the individual head shape on the sound field have to be taken into account. Achieving such a 6DOF reproduction without a priori knowledge of the room and its acoustic properties still remains a huge challenge. Improving the psychoacoustic models of room perception, perception of complex environments, and specific content to identify potentials for physical simplification without affecting the quality of the illusion is an inevitable step in solving this task.
